# Antimicrobial Susceptibility of Clinical Oral Isolates of *Actinomyces* spp.

**DOI:** 10.3390/microorganisms10010125

**Published:** 2022-01-07

**Authors:** Alexandra Wolff, Arne C. Rodloff, Paul Vielkind, Toralf Borgmann, Catalina-Suzana Stingu

**Affiliations:** Institute for Medical Microbiology and Virology, University Hospital, University of Leipzig, 04103 Leipzig, Germany; acr@medizin.uni-leipzig.de (A.C.R.); gti-paule88@gmx.de (P.V.); toralf_borgmann@hotmail.com (T.B.); CatalinaSuzana.Stingu@medizin.uni-leipzig.de (C.-S.S.)

**Keywords:** *Actinomyces*, susceptibility testing, agar dilution, E-Test

## Abstract

*Actinomyces* species play an important role in the pathogenesis of oral diseases and infections. Susceptibility testing is not always routinely performed, and one may oversee a shift in resistance patterns. The aim of the study was to analyze the antimicrobial susceptibility of 100 well-identified clinical oral isolates of *Actinomyces* spp. against eight selected antimicrobial agents using the agar dilution (AD) and E-Test (ET) methods. We observed no to low resistance against penicillin, ampicillin-sulbactam, meropenem, clindamycin, linezolid and tigecycline (0–2% ET, 0% AD) but high levels of resistance to moxifloxacin (93% ET, 87% AD) and daptomycin (83% ET, 95% AD). The essential agreement of the two methods was very good for benzylpenicillin (EA 95%) and meropenem (EA 92%). The ET method was reliable for correctly categorizing susceptibility, in comparison with the reference method agar dilution, except for daptomycin (categorical agreement 87%). Penicillin is still the first-choice antibiotic for therapy of diseases caused by *Actinomyces* spp.

## 1. Introduction

*Actinomyces* species are Gram-positive, anaerobic bacteria that are colonizing the skin, gastrointestinal and genitourinary tract. They play an important role in the maturation of dental plaque and pathogenesis of periodontitis [1,2,3,4].

They are also associated with infections such as actinomycosis, cerebral or oral abscesses, infections of the eyes, ear, nose and throat, and pulmonary infections [5,6,7,8,9,10,11,12,13,14,15]. Actinomycosis is a rare and chronic disease defined by its anatomical location. The cervicofacial actinomycosis represents the most prevalent type, followed by thoracic, abdominal and pelvic infection [16,17,18,19,20]. The disease is often misdiagnosed because of mimicking other infections or malignancy and is therefore inappropriately treated [21,22,23]. The treatment is carried out with high-dose parental and oral β-lactam antibiotics for an extended period and in some cases surgical debridement is needed [18,20]. The clinical significance of many newly described *Actinomyces* spp. has yet to be proven, but some species are possibly associated with polymicrobial infections in superficial soft-tissue abscesses [24,25,26].

Very limited data about the antimicrobial susceptibility of *Actinomyces* are available. *Actinomyces* spp. have been described over the years as susceptible to many antibiotics. However, the most recent literature reported increased resistance patterns in *Actinomyces* spp. and genetically closely related species of the order Actinomycetes, such as *Streptomyces*, generally being multi-drug-resistant, and newly described resistance mechanisms, such as rifampin-inactivating mechanisms, also have been found [27,28]. Other studies have determined that isolates of *A. europaeus* and *A. urogenitalis* showed resistance to piperacillin-tazobactam, cefriaxone, linezolid and clindamycin, respectively [29,30,31].

Due to their fastidious growth, routine laboratory identification and antimicrobial susceptibility testing can be challenging and thus not usually performed. Currently, there is no standardization for susceptibility testing of *Actinomyces* spp. and no recommended method. The agar dilution method represents the *gold standard* for susceptibility testing of anaerobes, although it is time-consuming and laborious. It also requires trained personnel, regular quality control and special equipment that not every routine laboratory provides.

Without periodic susceptibility testing one may oversee a shift in their resistance patterns. Moreover, the interpretation of these data may be hampered by difficulties in the accurate identification of *Actinomyces* spp. and sometimes the lack of use of standardized susceptibility testing methods.

As antibiotic resistance within the Actinomycetes is emerging, the aim of the study was to analyze the antimicrobial susceptibility of well-identified clinical oral isolates of *Actinomyces* spp. using agar dilution and the E-Test method.

## 2. Materials and Methods

### 2.1. Bacterial Isolates

A total of 100 clinical oral isolates of *Actinomyces* spp. were used in this study. They originated from periodontal pockets, supra and subgingival plaque of 20 patients with chronic periodontitis and 15 healthy subjects, as described previously [1]. The initial selection of the clinical strains was determined based on colony and Gram-stain morphology, pigmentation and biochemical methods such as the CAMP test and catalase test.

The species included in this study reflect the diversity of the *Actinomyces* genus in the oral cavity. Since not all oral *Actinomyces* species are present in the database of commercial identification kits (Rapid ID 32 A, API Coryne, VITEK 2, ANC ID Card, bioMerieux (Marcy-l’Étoile, France), and VITEK-MS, bioMerieux), the identification based on existing data is challenging. The Identification was performed by matrix-assisted laser desorption/ionization time-of-flight mass spectrometry (MALDI TOF MS) (28 strains) using a new in-house developed identification software under the protocol of T. Borgmann, or by 16s ribosomal RNA (72 strains) [32]. The strain sequences were compared with sequences deposited in the Human Oral Microbiome Database (HOMD) using the program HOMD 16S rRNA Sequence Identification.

All the isolates were stored at −80 °C in skim milk. Prior to susceptibility testing the strains were cultivated on Brucella blood agar (Thermo fisher Scientific, Oxoid Microbiology Products, Hampshire, UK) at 37 °C in an anaerobic chamber containing 15% CO_2_, 5% H_2_ and 80% N_2_ (Whitley MG 1000, anaerobic workstation, Meintrup Laborgeraete GmbH, Laehden-Holte, Germany).

### 2.2. Antibiotics

In our study, we tested the minimum inhibitory concentration (MIC) of penicillin (PEN), ampicillin-sulbactam (SAM), meropenem (MRP), clindamycin (CD), daptomycin (DAP), moxifloxacin (MXF), linezolid (LNZ) and tigecycline (TGC). Penicillin is the antibiotic of first choice for clinical infections with *Actinomyces* spp. Ampicillin/sulbactam combines a β-lactam antibiotic with a β-lactamase inhibitor used against penicillin-resistant bacteria producing β-lactamase. Clindamycin is used against infections with Gram-positive bacteria and is effective against anaerobes. Furthermore, it is an antibiotic of first choice to treat patients with a penicillin allergy. Clindamycin and moxifloxacin are often prescribed in dentistry, and we wanted to determine whether *Actinomyces* spp. developed resistance. Meropenem is a broad-spectrum antibiotic used against mixed infections that can usually be found in the oral cavity. Daptomycin is a highly effective reserve antibiotic against skin and soft tissue infections caused by Gram-positive bacteria. Tigecycline is a reserve antibiotic with a broad spectrum, including Gram-positive, Gram-negative and multidrug-resistant bacteria in severe infections. Linezolid is also a reserve antibiotic and is used against Gram-positive bacteria. Due to the lack of susceptibility data for *Actinomyces* spp., we chose to test linezolid in this study.

### 2.3. Inoculum Preparation

The MICs were obtained using the agar dilution method and E-Test methodology. All tests were performed using Brucella blood agar, containing 5% defibrinated sheep blood, which was incubated anaerobically for 48–72 h. Both methods were based on the European Committee for Antimicrobial Susceptibility Testing (EUCAST) standard procedure, which is as follows: The in-house-produced plates had an agar depth of 4 ± 0.5 mm. The plates were used within 3 days for the E-Test method and 8 h for the agar dilution method. We used a sterile cotton swab to pick colonies from an anaerobic overnight culture, then suspended in saline until an even turbidity of 1 McFarland was reached. After inoculation of the agar plates, we incubated them within 15 min in an anerobic chamber. Quality control (QC) was also conducted according to EUCAST recommendations. The cut-off values used for MIC testing were as follows: Benzylpenicillin sensitive (S) ≤ 0.25 mg/L, resistant (R) > 0.5 mg/L; ampicillin/Sulbactam S ≤ 4 mg/L, R > 8; meropenem S ≤ 2 mg/L, R > 8 mg/L; clindamycin S ≤ 4 mg/L, R > 4 mg/L; moxifloxacin PK-PD S ≤ 0.25 mg/L, R > 0.25 mg/L; tigecycline PK-PD S ≤ 0.5 mg/L, R > 0.5 mg/L; and linezolid PK-PD S ≤ 2 mg/L, R > 2 mg/L. For daptomycin there were no clinical breakpoints nor PK-PD breakpoints available at this time, so we postulated R ≥ 4 mg/L for this study.

#### 2.3.1. E-Test (ET) Method

Freshly prepared bacterial suspensions (1 McFarland) as described above were uniformly spread on the surface of Brucella blood agar plates (150 mm in diameter) using a sterile cotton swab. E-Test strip application was carried out in accordance with the manufacturer’s guidelines (BioMérieux, Lyon, France). After anaerobic incubation, the MICs were read and visually compared, following the EUCAST guidelines for anaerobic bacteria [33].

#### 2.3.2. Agar Dilution (AD) Method

Doubling stock solutions from each antimicrobial agent except tigecycline were prepared as described in the EUCAST definitive document E.def 3.1 [34]. Serial dilutions were prepared, and each added to Brucella blood agar under sterile conditions. The agar plates with a concentration of 0.03125–16 mg/L (clindamycin), 0.125–8 mg/L (daptomycin), 0.03125–24 mg/L (meropenem, ampicillin-sulbactam), 0.06–4 mg/L (moxifloxacin), 0.03125–2 mg/L (penicillin) and 0.03125–8 mg/L (linezolid) were made and allowed to cool down and set.

A semiautomatic replicator device (A400 Multipoint inoculator, Bachofer GmbH, Germany) was used to inoculate the prepared bacterial suspensions onto the freshly manufactured agar plates. Once inoculated, the agar dilution plates had a final approximate inoculum size of 10^5^ CFU/spot.

As the growth controls for each test, we used Brucella blood agar with no antibiotics and the following quality control strains: *Actinomyces oris* DSM14222, *Actinomyces viscosus* DSM43798, *Actinomyces graevenitzii* DSM15540, *Actinomyces odontolyticus* DSM43331, *Actinomyces neuii* DSM8576, *Actinomyces gerencseriae* ATCC23860, *Actinomyces meyeri* MCCM01956, *Actinomyces israelii* ATCC12107 and *Actinomyces naeslundii* ATCC12109.

All the plates were placed into an anaerobic chamber within 15 min after inoculation and then incubated for 48–72 h under anaerobic conditions (Whitley MG 1000, anaerobic workstation, Meintrup Laborgeraete GmbH, Laehden-Holte, Germany). The results were read and visually compared following the European Committee for Antimicrobial Susceptibility Testing (EUCAST) guidelines for anaerobic bacteria [19]. 

### 2.4. Statistical Analysis

The MIC-Range, MIC_50,_ MIC_90_, geometric means and percentage of resistance in accordance with the EUCAST breakpoints were determined and compared. In the absence of EUCAST breakpoints for moxifloxacin, tigecycline, linezolid and daptomycin, we used the PK-PD breakpoints to interpret the results. For daptomycin we used an MIC of 4 mg/L as the cut-off value for this study. The essential agreement (EA) between the E-Test and agar dilution methods was determined using the percentage of isolates where the ET MIC was within ±1.0 log_2_ dilution of the reference AD MIC value for each agent tested. The categorical agreement (CA) was also calculated, being defined as the percentage of isolates tested that yielded the same categorical interpretation as the reference method. CA discrepancies are subdivided as follows: very major error (VME), major error (ME) and minor error (mE). An adequate susceptibility testing system is expected to have an EA and CA ≥ 90%, and the acceptable error rates are VME ≤ 1.5%, ME < 3% and mE ≤ 10% [21].The descriptive statistics were performed using SPSS (IBM^®^ SPSS^®^ Statistics 26.0).

## 3. Results

Almost all isolates were susceptible to benzylpenicillin, ampicillin-sulbactam, meropenem, clindamycin, tigecycline and linezolid. In contrast, most of the *Actinomyces* isolates were resistant to moxifloxacin and showed high MIC values for daptomycin. The results for both methods varied little, showing higher resistance using the E-Test method. Number of isolates, categorical agreement (CA), very major error (VME), major error (ME) and minor error (mE) of the E-Test (ET) MICs compared to the agar dilution (AD) MICs of the selected antimicrobials for 100 isolates of *Actinomyces* spp. using EUCAST breakpoints or ECOFFS (v10.2) are shown in Table 1. Applying the criteria mentioned above, the E-Test method was reliable for correctly categorizing susceptibility, except for daptomycin, where there are no actual categorizing standards. Moxifloxacin yielded an unacceptably high percentage of errors of categorization. Therefore, the E-Test method did not meet the criteria for a reasonable susceptibility testing system for this antibiotic.

Values of MIC_50_, MIC_90_, the MIC range and the geometric means for 100 isolates of *Actinomyces* tested by the E-Test (ET) and agar dilution (AD) methods, as well as the calculated essential agreement between both methods, are shown in Table 2. Benzylpenicillin and meropenem yielded the highest EA with 95% and 92%, respectively. The EA of daptomycin was 81%. We observed major discrepancies in the EA for linezolid (56%), ampicillin-sulbactam, clindamycin (52% each) and moxifloxacin (50%). For linezolid and moxifloxacin, the ET MIC values were two 2-fold dilutions higher than the MIC values obtained by AD. For ampicillin-sulbactam, meropenem, clindamycin and benzylpenicillin the ET MIC values were predominantly a 2-fold dilution lower than those obtained by AD.

Table 3 shows the MIC ranges of representatives of eleven *Actinomyces* species tested in our study. For linezolid and moxifloxacin, the ET MIC values were two 2-fold dilutions higher than the MIC values obtained by AD. For ampicillin-sulbactam, meropenem, clindamycin and benzylpenicillin the ET MIC values were predominantly a 2-fold dilution lower than those obtained by AD. The poor growth of some isolates sometimes hampered the interpretation of the E-Test MIC. A single isolate of *A. odontolyticus* showed multi-drug resistance to benzylpenicillin, meropenem, moxifloxacin and daptomycin when using the E-Test method. This resistance pattern was confirmed by the agar dilution method for daptomycin, meropenem and penicillin, but not for moxifloxacin.

Table 4 shows the agar dilution and E-Test MIC range for the eight antimicrobial agents tested against the three most common *Actinomyces* species in our study. *A. oris* and *A. naeslundii* have similar MICs whereas the MICs for isolates of *A. odontolyticus* are 1 to 2 log2 dilutions higher for meropenem, moxifloxacin, benzylpenicillin, ampicillin-sulbactam, linezolid and tigecycline.

## 4. Discussion

In our study, we tested 100 clinical isolates of oral *Actinomyces* spp. against eight antibiotics by the agar dilution and E-Test methods.

Oral *Actinomyces* susceptibility testing is not usually performed, as this genus is known for its susceptibility to many antibiotics and for a lack of standardized testing methods. While various methods can be applied, the recommended methods for antimicrobial susceptibility testing of anaerobes are the disc diffusion method, broth microdilution method, agar dilution method and gradient tests, such as the E-Test method. The disc diffusion method is a convenient and easily performable technique but recommended for fast-growing anaerobes like *Bacteroides fragiles* strains. The broth microdilution method is a highly accurate method for testing several antibiotics simultaneously. The disadvantage is the inconsistent growth of anaerobe species, except *Bacteroides* spp. The agar dilution method, currently representing the *gold standard* for anaerobic bacteria, is accurate, and a high number of isolates can be tested simultaneously. It is a time-consuming and labor-intensive method recommended for specially equipped laboratories. The E-Test method is fast, cost-effective and easy to perform in routine laboratories and especially used for testing a small number of isolates or multiple antibiotics at once. The latter two methods were selected to ensure reliable results.

Numerous studies signaled an increasing resistance of anaerobes in the last years. Thus, assuming that the usually prescribed antibiotics are still highly effective is no longer appropriate. The resistance rates of *Actinomyces* spp. obtained with the agar dilution method are similar to those from other recently conducted surveys, regarding penicillin, carbapenems, clindamycin, linezolid, daptomycin and moxifloxacin [21,22,23,24,25,26]. We detected one isolate of *A. odontolyticus* that showed multi-drug resistance (MDR) to benzylpenicillin, meropenem, moxifloxacin and daptomycin. Another isolate of *A. gerencseriae* was resistant to clindamycin and ampicillin-sulbactam. Though MDR is uncommon among the *Actinomyces*, this is an increasing problem for other anaerobic bacteria. The literature refers to *Bacteroides fragilis* isolates with resistance to penicillin, piperacillin-tazobactam, meropenem, clindamycin and metronidazole [35,36,37,38]. *Clostridioides difficile* showed MDR for moxifloxacin, clindamycin, erythromycin and rifampicin. Furthermore, MDR was observed for *Prevotella* spp., *Finegoldia magna*, *Veillonella* spp. and *Cutibacterium acnes* [39]. The Actinomycetes order includes beside the genus *Actinomyces* other important genera, *Nocardia* and *Streptomyces*. These genera can develop a remarkable survival ability and can cause life-threatening infections. Reliable identification and susceptibility testing can contribute to reduced mortality [40,41]. Because of the high prevalence of *Actinomyces* spp. in polymicrobial infections, the emerging resistance patterns of anaerobe bacteria should be observed. Routine susceptibility testing is recommended and using a reliable and convenient method for testing a limited number of isolates is necessary for routine laboratories. However, only a few studies have focused on susceptibility testing of oral *Actinomyces* spp. Our study underlines the importance of performing susceptibility testing even though there are no guidelines for *Actinomyces* organisms. To interpret the results, we used for both methods the EUCAST breakpoints for Gram-positive anaerobes, when available, even though it does not specify any method to be used with them. In absence of them, we used the PK-PD (non-species related) breakpoints.

The fastidious character of *Actinomyces* spp. leads to difficulties in cultivation and identification. Consequently, this can explain some discrepancies between the MIC values obtained using the E-Test and agar dilution methods. The poor and slow growth of some isolates causing diffuse edges of their inhibition zones or the growing of only few colonies makes reading the MICs more difficult and may result in lower MIC values, such as those for ampicillin-sulbactam, meropenem, clindamycin and benzylpenicillin.

Our in vitro data suggest that oral *Actinomyces* spp. are susceptible to benzylpenicillin, ampicillin-sulbactam, meropenem, clindamycin, linezolid and tigecycline. Our findings are in line with other recently conducted surveys covering oral *Actinomyces* species [30,31,42,43,44,45]. In general, there were no differences regarding susceptibility within the different *Actinomyces* species.

Essentially, the high effectiveness of β-lactam antibiotics, linezolid and tigecycline correspond to other studies [31,46,47], although some studies have found higher resistance rates, from 16–34%, for clindamycin for the *Actinomyces* spp. tested [48]. The resistance against moxifloxacin was confirmed by other studies [47,49]. Because there are no guidelines or PK-PD breakpoints for daptomycin, we cannot prove the efficacy of this antibiotic. It is noticeable that the MICs for daptomycin where very high in comparison with the other antibiotics used in this study. Our results are similar to those of another study: MIC_50_ 8 mg/L and MIC_90_ 32 mg/L [50]. Therefore, it may be assumed that *Actinomyces* spp. are resistant to daptomycin.

In this study, we showed that the E-Test method could be a promising susceptibility testing alternative to the agar dilution method, especially for ß-lactams for routine laboratory testing.

## 5. Conclusions

Although *Actinomyces* are susceptible to many antibiotics, such as β-lactams, meropenem and reserve antibiotics such as clindamycin linezolid and tigecycline, the empirical use of some antimicrobial agents such as moxifloxacin or daptomycin, especially in polymicrobial infections, could lead to a failed treatment. The constant threat of increased resistance and MDR in anaerobic bacteria needs to be monitored. These data underline the importance of routine susceptibility testing in laboratories using reliable methods, such as the E-Test method, to provide the relevant information to clinicians and to prevent treatment failure.

## Figures and Tables

**Table 1:** Number of susceptible (S), reduced susceptible (>S, <R) and resistant (R) *Actinomyces* spp. isolates against eight antimicrobial agents tested by the E-Test (ET) and agar dilution (AD) methods, using EUCAST breakpoints or ECOFFS (v10.2).

	ET	AD	ET	AD	ET	AD				
**Antibiotic**	**S (*n*)**		**>S, <R (*n*)**		**R (*n*)**		**CA (%)**	**VME (%)**	**ME (%)**	**mE (%)**
Benzylpenicillin	98	99	0	1	2	0	98	0	1	1
Ampicillin-Sulbactam	99	100	1	0	0	0	100	0	0	1
Clindamycin	99	100	0	0	1	0	99	0	1	0
Meropenem	98	99	1	1	1	0	99	0	0	2
Moxifloxacin										
Moxifloxacin PK-PD	7	13	0	0	93	87	90	2	8	0
Linezolid										
Linezolid PK-PD	99	100	1	0	0	0	100	0	0	1
Daptomycin										
Daptomycin PK-PD					(83 *)	(95 *)	n.a	n.a	n.a	n.a
Tigecycline		n.a		n.a		n.a	n.a			
Tigecycline PK-PD	99	n.a	0	n.a	1	n.a				

(*) Number of isolates tested with an MIC ≥ 4 mg/L. Abbreviations: n.a = data not available.

**Table 2:** MIC_50_, MIC_90_, geometric mean, resistance rate (R%) and EA (%) of the tested antibiotics, compared using the ET and AD methods.

		Method		
		ET	AD	EA(%)
Antibiotic		MIC (mg/L)		
Clindamycin	range mg/L	<0.03125–64	0.0625–1	52
	MIC_50_	0.5	1	
	MIC_90_	1	1	
	Mean	1.11603125	0.894375	
	R%	1	0	
Daptomycin	range mg/L	<0.03125–64	2–>8	81
	MIC_50_	16	>8	
	MIC_90_	32	>8	
	Mean	15.24781	7.68	
	R%	(83 *)	(95 *)	
Meropenem	range mg/L	<0.03125–>32	0.03125–4	92
	MIC_50_	0.03125	0.0625	
	MIC_90_	0.125	0.0625	
	Mean	0.424688	0.060921717	
	R%	1	0	
Moxifloxacin	range mg/L	<0.03125–32	0.125–>4	50
	MIC_50_	2	1	
	MIC_90_	16	2	
	Mean	5.601875	1.18	
	R%	93	84	
Penicillin	range mg/L	<0.03125–1	<0.0625–0.5	95
	MIC_50_	0.03125	0.0625	
	MIC_90_	0.125	0.125	
	Mean	0.08375	0.07625	
	R%	2	0	
Ampicillin-Sulbactam	range mg/L	<0.03125–8	<0.03125–2	52
	MIC_50_	0.03125	0.125	
	MIC_90_	0.125	0.5	
	Mean	0.195313	0.259375	
	R%	0	0	
Linezolid	range mg/L	<0.03125–4	0.125–1	56
	MIC_50_	0.5	0.125	
	MIC_90_	1	0.25	
	Mean	0.491875	0.20625	
	R%	0	0	
Tigecycline	range mg/L	<0.03125–1	n.a	n.a
	MIC_50_	0.0625	n.a	
	MIC_90_	0.125	n.a	
	Mean	0.073125	n.a	
	R%	1	n.a	

(*) Number of isolates tested with an MIC ≥ 4 mg/L. Abbreviations: n.a = data not available.

**Table 3:** MIC range of *Actinomyces* spp. tested against selected antimicrobial agents comparing the agar dilution and E-Test methods.

Antibiotic	CD		DAP		MRP	
Method	ET	AD	ET	AD	ET	AD
Species (No. of Isolates Tested)	MIC (mg/L)		MIC (mg/L)		MIC (mg/L)	
*A. oris* (*n* = 20)	0.03125–2	0.5–1	1–32	2–>8	<0.03125	0.03125–0.0625
*A. naeslundii* (*n* = 33)	<0.03125–1	0.25–1	0.5–32	4–>8	<0.03125–0.125	0.03125–0.0625
*A. odontolyticus* (*n* = 18)	0.03125–1	0.125–1	0.25–64	2–>8	<0.03125–>32	0.03125–4
*A. gerencseriae* (*n* = 8)	0.0625–64	1	0.5–32	4–>8	<0.03125	0.03125–0.0625
*A. graevenitzii* (*n* = 4)	0.125–4	0.5–1	16–32	>8	0.125	0.03125–0.0625
*A. israelii* (*n* = 4)	<0.03125–0.0625	0.125–1	<0.03125–4	2–>8	<0.03125	0.03125–0.125
*A. meyeri* (*n* = 3)	0.5–1	1	32–64	>8	0.125–0.25	0.0625
*A. neuii* (*n* = 2)	<0.03125	0.0625–0.125	1	2	0.125	0.0625
*A. johnsonii* (*n* = 4)	0.125–1	1	8–16	8–>8	<0.03125–0.0625	0.03125–0.0625
*A. massiliensis* (*n* = 3)	0.25–2	1	2–4	8–>8	<0.03125	0.03125–0.0625
*A. timonensis* (*n* = 1)	0.25	1	32	>8	<0.03125	0.0625
Total Range in mg/L	<0.03125–64	0.0625–1	<0.03125–64	2–>8	<0.03125–>32	0.03125–4
**Antibiotic**	**MXF**		**PEN**		**SAM**	
**Method**	**ET**	**AD**	**ET**	**AD**	**ET**	**AD**
**Species (No. of Isolates Tested)**	**MIC (mg/L)**		**MIC (mg/L)**		**MIC (mg/L)**	
*A. oris* (*n* = 20)	0.25–8	0.125–2	<0.03125–0.125	0.0625–0.125	<0.03125–0.125	<0.03125–0.5
*A. naeslundii* (*n* = 33)	0.125–>32	0.25–>4	<0.03125–0.25	0.03125–0.25	<0.03125–0.5	<0.03125–0.5
*A. odontolyticus* (*n* = 18)	0.5–32	0.25–4	<0.03125–1	0.0625–0.5	<0.03125–4	0.125–2
*A. gerencseriae* (*n* = 8)	0.25–>32	0.25–4	<0.03125–0.125	0.0625–0.125	<0.03125–8	<0.03125
*A. graevenitzii* (*n* = 4)	8–16	2	0.0625	0.03125–0.0625	0.0625–0.125	0.125–0.5
*A. israelii* (*n* = 4)	<0.03125–4	0.125–2	<0.03125	0.0625	<0.03125–0.0625	<0.03125–1
*A. meyeri* (*n* = 3)	4–16	1	0.125	0.0625	0.0625–0.125	0.25–0.5
*A. neuii* (*n* = 2)	1	0.25	0.25–1	0.0625	0.5	1
*A. johnsonii* (*n* = 4)	1–4	1	<0.03125	0.0625	<0.03125–0.0625	<0.03125–0.25
*A. massiliensis* (*n* = 3)	0.25–1	0.25–1	<0.03125	0.0625	<0.03125	<0.03125–0.125
*A. timonensis* (*n* = 1)	<0.03125	2	<0.03125	0.0625	<0.03125	0.125
Total Range in mg/L	<0.03125–>32	0.125–>4	<0.03125–1	0.03125–0.5	<0.03125–8	<0.03125–2
**Antibiotic**	**LNZ**		**TGC**			
**Method**	**ET**	**AD**	**ET**	**AD**		
**Species (No. of Isolates Tested)**	**MIC (mg/L)**		**MIC (mg/L)**			
*A. oris* (*n* = 20)	0.125–1	0.125–0.5	<0.03125–0.125	n.a		
*A.naeslundii* (*n* = 33)	<0.03125–2	0.125–0.5	<0.03125–0.125	n.a		
*A. odontolyticus* (*n* = 18)	0.25–4	0.125–1	<0.03125–1	n.a		
*A. gerencseriae* (*n* = 8)	0.0625–1	0.125–0.25	<0.03125–0.0625	n.a		
*A. graevenitzii* (*n* = 4)	0.5–1	0.125	0.125–0.5	n.a		
*A. israelii* (*n* = 4)	<0.03125–0.5	0.125–0.5	<0.03125	n.a		
*A. meyeri* (*n* = 3)	0.5–1	0.125	0.0625–0.125	n.a		
*A. neuii* (*n* = 2)	0.25–0.5	0.125	<0.03125	n.a		
*A. johnsonii* (*n* = 4)	0.25–0.5	0.125–0.25	<0.03125–0.0625	n.a		
*A. massiliensis* (*n* = 3)	0.125–0.25	0.125	<0.03125	n.a		
*A. timonensis* (*n* = 1)	<0.03125	0.25	<0.03125	n.a		
Total Range in mg/L	<0.03125–4	0.125–1	<0.03125–1	n.a		

Abbreviations: n.a = data not available.

**Table 4:** Comparison of agar dilution MICs and E-Test MICs by antimicrobial agent tested against the three most common Actinomyces species in our study.

		MIC (mg/L)					
		ET			AD		
Species (No. of Tested Isolates)	Antibiotic	Range	MIC_50_	MIC_90_	Range	MIC_50_	MIC_90_
*A. oris*	CD	0.03125–2	0.25	1	0.5–1	1	1
(*n* = 20)	DAP	1–32	8	16	2–>8	>8	>8
	MRP	<0.03125–0.0625	<0.03125	<0.03125	0.03125–0.0625	0.0625	0.0625
	MXF	0.25–8	2	4	0.125–2	0.5	1
	PEN	<0.03125–0.125	<0.03125	0.125	0.0625–0.125	0.0625	0.0625
	SAM	<0.03125–0.125	0.0625	0.125	<0.03125–0.5	0.125	0.5
	LNZ	0.125–1	0.25	0.5	0.125–0.5	0.125	0.25
	TGC	<0.03125–0.125	0.0625	0.0625	n.a	n.a	n.a
*A. neaslundii*	CD	<0.03125–1	0.5	1	0.25–1	1	1
(*n* = 33)	DAP	0.5–32	16	16	4–>8	>8	>8
	MRP	<0.03125–0.125	<0.03125	<0.03125	0.03125–0.0625	0.03125	0.0625
	MXF	0.125->32	2	2	0.25–>4	1	1
	PEN	<0.03125–0.25	<0.03125	0.125	0.03125–0.25	0.0625	0.0625
	SAM	<0.03125–0.5	<0.03125	0.125	<0.03125–0.5	<0.03125	0.25
	LNZ	<0.03125–2	0.25	0.5	0.125–0.5	0.125	0.25
	TGC	<0.03125–0.125	0.0625	0.0625	n.a	n.a	n.a
*A. odontolyticus*	CD	0.03125–1	0.5	1	0.125–1	1	1
(*n* = 18)	DAP	0.25–64	32	32	2–>8	>8	>8
	MRP	<0.03125–>32	0.125	0.5	0.03125–4	0.0625	0.25
	MXF	0.5–32	8	16	0.25–>4	2	2
	PEN	<0.03125–1	0.125	0.125	0.0625–0.5	0.0625	0.125
	SAM;	<0.03125–4	0.125	0.25	0.125–2	0.5	1
	LNZ	0.25–4	0.5	1	0.125–1	0.125	0.5
TGC		<0.03125–1	0.0625	0.125	n.a	n.a	n.a

Abbreviations: n.a = data not available.

## Data Availability

Not applicable.
